# Age-Related Attenuation of Dominant Hand Superiority

**DOI:** 10.1371/journal.pone.0000090

**Published:** 2006-12-20

**Authors:** Tobias Kalisch, Claudia Wilimzig, Nadine Kleibel, Martin Tegenthoff, Hubert R. Dinse

**Affiliations:** 1 Institute for Neuroinformatics, Neural Plasticity Lab, Department of Theoretical Biology, Ruhr-University Bochum Bochum, Germany; 2 California Institute of Technology, Division of Biology Pasadena, California, United States of America; 3 Department of Neurology, BG-Kliniken Bergmannsheil, Ruhr-University Bochum Bochum, Germany; University of Birmingham, United Kingdom

## Abstract

**Background:**

The decline of motor performance of the human hand-arm system with age is well-documented. While dominant hand performance is superior to that of the non-dominant hand in young individuals, little is known of possible age-related changes in hand dominance. We investigated age-related alterations of hand dominance in 20 to 90 year old subjects. All subjects were unambiguously right-handed according to the Edinburgh Handedness Inventory. In Experiment 1, motor performance for aiming, postural tremor, precision of arm-hand movement, speed of arm-hand movement, and wrist-finger speed tasks were tested. In Experiment 2, accelerometer-sensors were used to obtain objective records of hand use in everyday activities.

**Principal Findings:**

Our data confirm previous findings of a general task-dependent decline in motor performance with age. Analysis of the relationship between right/left-hand performances using a laterality index showed a loss of right hand dominance with advancing age. The clear right-hand advantage present at younger ages changed to a more balanced performance in advanced age. This shift was due to a more pronounced age-related decline of right hand performance. Accelerometer-sensor measurements supported these findings by demonstrating that the frequency of hand use also shifted from a clear right hand preference in young adults to a more balanced usage of both hands in old age. Despite these age-related changes in the relative level of performance in defined motor tasks and in the frequency of hand use, elderly subjects continued to rate themselves as unambiguous right-handers.

**Conclusion:**

The discrepancy between hand-specific practical performance in controlled motor tests as well as under everyday conditions and the results of questionnaires concerning hand use and hand dominance suggests that most elderly subjects are unaware of the changes in hand dominance that occur over their lifespan, i.e., a shift to ambidexterity.

## Introduction

The hand-arm system is the most active part of the human upper extremities. Over the lifespan, hands undergo many physiological and anatomical changes [1] where both intrinsic and extrinsic factors contribute to age-related alterations. For example, muscle mass and strength decrease, especially after the age of sixty years [2]. Other age-related changes include decreased abilities to maintain steady forces [3], [4], an increase in time required to manipulate small objects [5], and a clear decrease in finger-pinch strength [6]. With increasing age, a decline of hand movement coordination occurs [7] which can lead to an impaired ability to perform everyday activities [8].

A decline in hand function can result from changes in the peripheral nervous system such as decreased nerve conduction velocity, sensory perception, or excitation-contraction coupling of motor units [9], [10]. It has been suggested that the higher muscle fatigue resistance typically found in the elderly was attributable to differences in both the muscle and the central nervous system [11]. Moreover, impairment of sensory perception is thought to be a key component of decreased fine motor functioning [12]. Besides age-related changes in the sensory motor system, it remains unclear how environmental factors such as declining physical activity associated with aging [13], [14] and sedentary lifestyles contribute to impaired hand function [15], [16].

While a general age-related decline in hand performance and hand function is undisputed, little is known about possible changes in hand dominance, i.e. about asymmetries of hand use that develop with advancing age. Several questionnaires like the “Edinburgh Handedness Questionnaire” [17], “Revised Waterloo Handedness Questionnaire” [18], “Annett handedness questionnaire” [19] as well as practical tests such as the “WatHand Box Test” [20], “Jebsen Test of Hand Function” [21], tapping-tasks and pegboard tests are available to assess hand dominance. These two approaches differ in that questionnaires only detect subjective preferences towards the use of the dominant or non-dominant hand in specified situations, but not necessarily the level of hand performance itself [22]. However, there is agreement that in young healthy subjects self-rated hand dominance and the level of motor performance is highly correlated [23], [24], [25].

We addressed the question of possible changes in hand dominance with advancing age in elderly subjects 65 to 90 years of age. Handedness can be defined as the preference or hand-difference in task performance [22]. To analyze both factors, we combined self-rating with questionnaires with objective measurements of hand dominance using a conventional fine motor test-series (Experiment 1). This provided insight into how the performance of each hand is differentially affected by age. Additionally, sensors were used to record the frequency of hand use during everyday activities which was compared with self-rated hand dominance (Experiment 2). We found that the superior performance of the dominant hand present at younger ages is progressively lost with advancing age due to a more pronounced age-related decline of hand function in the dominant hand. Interestingly, according to the subjective self-rated questionnaire, the older subjects were mostly unaware of these changes.

## Methods

Elderly subjects in senior residences were recruited by poster announcements or by word of mouth. All subjects were tested by a clinical neurologist to determine that they were without neurological symptoms and in good physical condition. Eligibility criteria were lucidity, independence in everyday activities, and the absence of motor handicaps such as functional impairment due to arthritis or other causes of joint immobility. Subjects with significant visual or hearing loss, cerebro-vascular or spinal diseases, pathological tremor, or any functional limitations of the upper limbs as a consequence of stroke or Parkinson's disease were excluded from the study. Medication taken by the subjects was documented to prevent the influence of drugs that may affect the central nervous system. An assessment of cognitive abilities was made using the “Mini Mental State Examination” [26]. Only persons with scores of 27 to 30 out of 30 possible points (indicative of “no dementia”) participated in the study. Accordingly, the subjects included in our study represent a subpopulation clearly biased towards mental and physical fitness. Young subjects were recruited by poster announcements from the university community. These individuals reported no known neurological disorders.

Hand preference was determined throughout the study with the “Edinburgh Handedness Inventory” (EHI) [17] which classifies handedness on the basis of a short interview on hand preference in the performance of routine practical tasks. The questionnaire evaluates handedness values from –100 for extreme left hand use to +100 for extreme right hand use. Only persons with unambiguous right hand dominance (≥ +70 points) and without a history of hand switching during their lifetime were included. The study was performed in accordance with the Declaration of Helsinki. Subjects gave written informed consent, and the protocol was approved by the local ethics committee of the Ruhr-University Bochum.

### Experiment 1

Sixty healthy volunteers (34 females and 26 males) participated in this study. Their self-rated handedness (EHI) was compared to the computer-based assessment of their dexterity. Subjects were divided into four age groups designated “25,” “50,” “70,” and “80” in accordance with the average age of the group. Group 25 included 14 subjects (9 females and 5 males) with a mean age of 24.8±3.1 years; group 50 included 14 subjects (8 females and 6 males) with a mean age of 51.8±3.2 years; group 70 included 18 subjects (9 females and 5 males) with a mean age of 70.9±2.7 years, and group 80 included 14 subjects (8 female and 6 males) with a mean age of 80.7±4.7 years.

Self-rated hand dominance revealed no significant differences between the four groups (Oneway ANOVA, F_(3,59)_ = 0.042, p = 0.989). The EHI scores were calculated as 85.00±7.60 for group 25, 83.08±9.58 for group 50, 83.89±9.48 for group 70, and 83.93±11.63 for group 80.

### Motor performance test-series

According to Fleishman [27], fine motor movements can be factorized with regard to speed, accuracy, and maintenance of upper limb positions. We investigated these aspects during execution of fine motor movements of the arms, hands, and fingers using the four separate tests described below.

**Figure 1:**
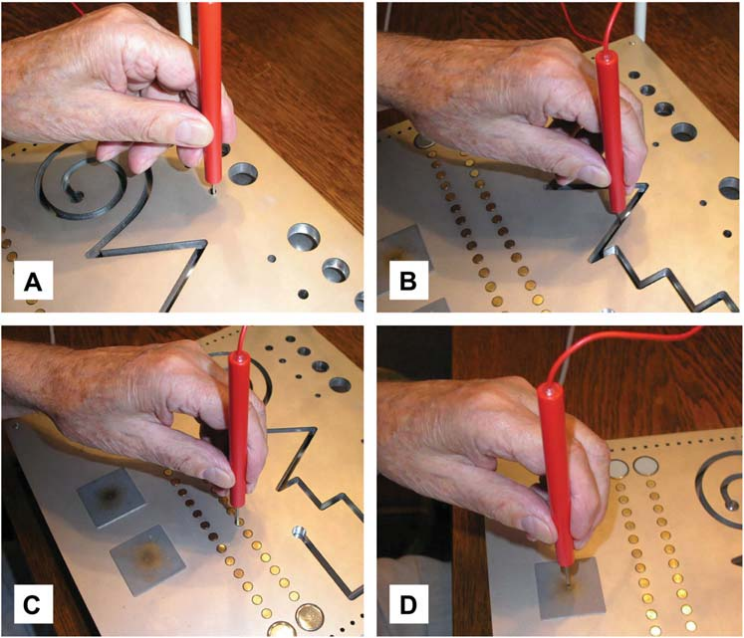
Examination of fine motor performance A commercial test-series was used to measure the fine motor performance of both hands. The “steadiness” task describes the ability to maintain prescribed arm-hand positions (**a**). “Line tracing” describes the ability to fulfil precise arm-hand movements (**b**). “Aiming” describes the ability to accomplish fast movements directed at small targets (**c**). Finally, “tapping” describes the ability to perform very fast, repetitive hand movements (**d**).

“Steadiness” (**Fig. 1a**) describes the ability to obtain a prescribed arm-hand position and to maintain it for a defined time period. “Line tracing” (**Fig. 1b**) describes the ability to fulfill precise, simultaneous arm-hand movements. “Aiming” (**Fig. 1c**) describes the ability to accomplish fast arm-hand movements for small targets. “Tapping” (**Fig. 1d**) describes the ability to perform very fast, repetitive wrist-finger movements with little emphasis on precision of movement.

All tests are available in the commercial test-series “MLS” (Dr. G. Schuhfried GmbH, Austria). The MLS is a computerized device for the accurate analysis of fine motor performance. Data registration was performed with the “Vienna-test-system” software, Version 5.05 (Dr. G. Schuhfried GmbH, Austria). We conducted a short form of the tests (10–15 minutes) in order to create a convenient test-situation for the elderly subjects.

The test board for the MLS can be used in both horizontal and vertical orientations. Two contact pencils are connected to the sides. The number and duration of contacts between pencils and test board are measured by closing electrical circuits (5V, 20mA). Data are transferred via an interface to a computer for analysis. The plain surface of the test board contains holes of different diameters, two rows of small contact plates, two large square contact plates, and a long groove.

Each task was explained by reading a standardized instruction sheet, and then the task was demonstrated to ensure that the subjects fully understood what they had to do. While the subjects sat in front of the board, support of the test arm was not permitted. All tests were performed with both the right and the left hands. To prevent systematic errors, subjects were randomly allocated to use the right or the left hand first.

#### Steadiness

The subject's task was to place the pencil into a small circular hole (5.8 mm) of the vertically positioned board, and hold it there without touching the edges for 32 seconds without support to steady the hand (**Fig. 1a**). This tested for the ability to hold a steady position, and for the absence of postural tremor [28]. Dependent variables were the number of errors, meaning the number of contacts the pencil made with the circumference the hole.

#### Line tracing

Subjects were instructed to insert the pencil perpendicular to the groove in the horizontally positioned board and follow its course without touching the edges (**Fig. 1b**). This tested ataxia and action tremor by assessing the ability to make visually-controlled, steady, guided movements [28]. Subjects were instructed to make as few errors as possible. Dependent variables were number of errors and the total time required to complete the task. Arm movements were carried out from the periphery to midline for each respective hand.

#### Aiming

Subjects had to consecutively hit each of a row of 20 linearly arranged small contact fields (diameter 5 mm, midpoint separation 9 mm) with the test pencil (**Fig. 1c**). This test assessed the degree of ataxia and the speed of movement by the ability to make rapid repeated aimed movements [28]. Again, the dependent variables were the number of errors (missed contact fields) and the total time needed to complete the task.

#### Tapping

Subjects were required to hit a square contact plate (40 by 40 mm) on the test board with the test pencil as frequently as possible (**Fig. 1d**). The measured parameter was number of hits achieved in a time interval of 32 seconds and thereby the speed of antagonistic oscillation [28]. Because, in this task, support of the forearm was allowed, the repetitive contacts had to be accomplished by wrist movements.

### Experiment 2

Another group of 36 healthy volunteers (16 females and 20 males) participated in the second experiment. This time, the self-rated handedness (EHI) was compared with the sensory-based assessment of hand use in everyday activities. As in the first experiment, the groups were designated according to the average age of the group. Group 25 included 13 subjects (6 females and 7 males) with a mean age of 27.3±4.8 years, group 50 had 9 subjects (3 females and 6 males) with a mean age of 52.4±3.1 years, and group 70 had 14 subjects (7 females and 7 males) with a mean age 72.9±3.6 years.

Self-rated hand dominance revealed no significant difference between the three groups (Oneway ANOVA, F_(2,35)_ = 0.051, p = 0.950). The EHI scores were 88.46±24.44 for group 25, 87.78±6.67 for group 50, and 88.87±10.27 for group 70.

### Assessment of hand movements in everyday activities

In order to obtain an objective measure of the use of the dominant and non-dominant hands in everyday activities, two ActiTrac® monitors (IM Systems Inc, USA) containing ceramic biaxial piezoelectric accelerometer sensors were used to record physical motion in two planes (vertical and front-to-back axes). The devices were fixed on the wrist of each hand, using belt-clips to allow unrestricted mobility of the subjects during recording, for several hours. The ActiTrac monitors measured acceleration at a rate of 40 Hz and accumulated the acceleration signals every 2 s resulting in 30 epochs per minute in units of mG (sensitivity 1.25 mG), which were stored for off-line analysis.

### Statistics

Data obtained for dominant and non-dominant hands in both experiments were analyzed using ANOVAs for the factors “GENDER” and “AGE-GROUP”, and repeated measures ANOVA designed for the factor “HAND”. In order to detect possible relationships between performance and age, single parameters were correlated with age (Pearson-correlation). To allow a direct comparison between the extent of age-dependency, correlation coefficients were Fisher-transformed and listed as Z-values. In order to discover possible changes in hand dominance, a laterality index (l−r)/(l+r) [l = left hand performance, r = right hand performance] was calculated based on the results obtained in the practical tests (Experiment 1), or hand use in everyday activities (Experiment 2). The indices describe the extent of hand dominance for a given task within a continuum ranging from 1 to −1 (left hand dominance). All indices were aligned so that positive values indicate right hand dominance within a given task. These indices were also correlated with age via Pearson correlations. All statistical analyses were calculated using SPSS version 12.0 (SPSS Inc, USA). A p value of <0.05 was considered significant.

## Results

### Experiment 1

#### Age-dependence of fine motor performance

**Figure 2:**
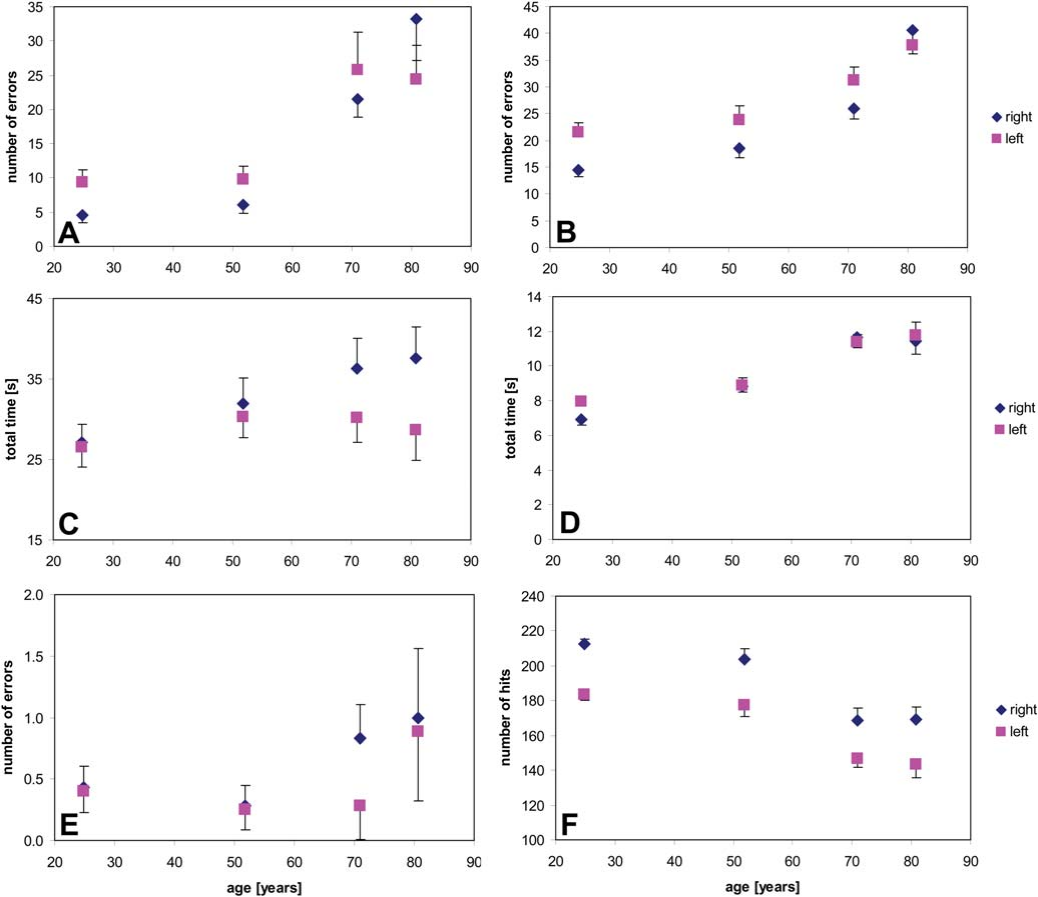
Age-related change of performance in fine motor tasks Group data illustrating right and left hand performance (±SEM) in “steadiness”, “line tracing”, “aiming” and “tapping” tasks. Linear Pearson's correlations revealed a significant influence of age on almost all parameters: steadiness (number of errors, **a**), line tracing (number of errors, **b**, total time right hand, **c**), aiming (total time, **d**) and tapping (number of hits, **f**) (p≤0.001). Only for line tracing (total time for the left hand, **c**), and aiming (number of errors, **e**) were a lack of age related influences found (p≥0.069).

For the majority of tasks tested, we found a clear decline in performance with increasing age. For the subtest “steadiness,” the number of contacts with the circumference of the hole increased significantly with age for the right-hand (r = 0.596, p<0.001; F_(3,52)_ = 14.421, p≤0.001) and left-hand (r = 0.414, p<0.001; F_(3,52)_ = 4.809, p = 0.005) executions of the task (**Fig. 2a**).

For the subtest “line tracing” measuring the precision of hand movements, the number of errors showed a significant increase with age for the right-hand (r = 0.625, p<0.001; F_(3,52)_ = 16.831, p≤0.001) and left-hand performances (r = 0.539, p<0.001; F_(3,52)_ = 8.030, p≤0.001) (**Fig. 2b**). Differences in the total time needed to fulfill the task reached significance for right hand performance (r = 0.275, p =  0.034; F_(3,52) = _1.738, p = 0.171), but not for left hand performance (r = 0.040, p = 0.761; F_(3,52) = _0.262, p = 0.853) (**Fig. 2c**).

For the subtest “aiming,” measuring the precision of target directed movements, the total time increased with age both for the right-hand (r = 0.640, p<0.001; F_(3,52) = _14.463, p≤0.001) and left-hand performances (r = 0.598, p≤0.001; F_(3,52) = _11.46, p≤0.001) (**Fig. 2d**). The number of errors failed to reach significance for right (r = 0.209, p = 0.109; F_(3,52)_ = 0.718, p = 0.546) and left hand performances (r = 0.236, p = 0.069; F_(3,52) = _3.603, p = 0.019) (**Fig. 2e**).

The performance of males and females differed only in the tapping task. In general, women made fewer contacts in 32 seconds irrespective of whether the task was performed with the right (F_(1,52)_ = 15.597, p≤0.001) or the left hand (F_(1,52)_ = 7.747, p = 0.007). This gender-specific difference in tapping performance was independent of the subject's age (non-significant interaction AGE*GENDER p≥0.277)

#### Laterality indices and age

To obtain a quantitative measure for the degree of right- or left-hand dominance, we calculated laterality indices ranging from −1 for left-hand superiority to +1 for right-hand superiority for each task and parameter. To find out if laterality changes with age, we performed linear correlation analyses between individual laterality indices averaged over all measured MLS parameters. We found a significant negative correlation (r = −0.406, p≤0.001) indicating a clear shift from right-hand superiority at the younger age towards a balanced performance at the older ages. In the younger age group, only 2 of 14 (14.3%) subjects showed left-hand superiority in average task performance, and 3 of 14 (21.4%) in group 50. However, among the individuals in groups 70 and 80, 13 of 32 subjects (41.6%) showed left-hand superiority. Accordingly, the difference between right- and left-hand performances, i.e. the dominance of the right hand, is largely abolished in the higher age groups. This results in an equalization of the level of performance of both hands, although, according to the Edinburgh questionnaire (EHI), all subjects were unambiguously right-handed (score ≥ +70).

**Figure 3:**
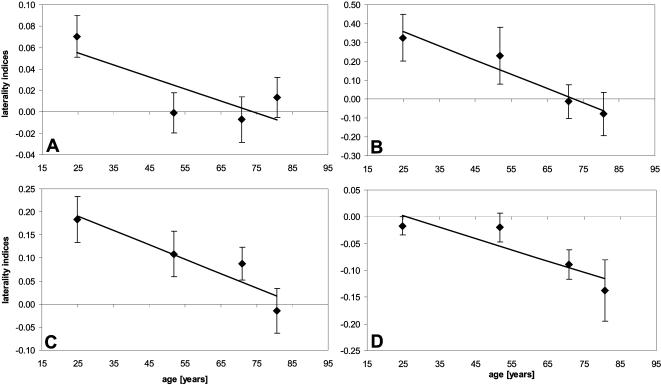
Laterality indices for fine motor performance Averaged laterality indices (±SEM) for the total time in the aiming task (a), number of errors in the steadiness task (b), the line tracing task (c), and the total time needed for the line tracing task (d).

To find out which tasks and parameters are most sensitive to age-related alterations in hand dominance, we analyzed the laterality indices separately (**Tab. 1**). According to this analysis, 4 of 6 laterality indices showed a significant correlation with age (**Fig. 3**): The total time for the aiming task (r = −0.286, p = 0.027) (**Fig. 3a**), the number of errors in the steadiness task ( r = −0.317, p = 0.014) (**Fig. 3b**), and the number of errors (r = −0.313, p = 0.015) (**Fig. 3c**) and total time (r = −0.324, p = 0.012) for the line tracing task (**Fig. 3d**) showed significant correlations with age. Tapping was the only task that showed no indication of change in hand superiority with age. For all tasks and conditions that showed changes in hand superiority, we did not observe any gender-specific differences of the laterality indices (F_(1,59)_≤2.234, p≥0.140).

**Table 1:**
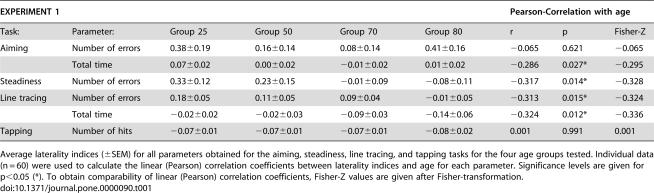
Laterality indices of fine motor performance in different age groups

EXPERIMENT 1						Pearson-Correlation with age		
Task:	Parameter:	Group 25	Group 50	Group 70	Group 80	r	p	Fisher-Z
Aiming	Number of errors	0.38±0.19	0.16±0.14	0.08±0.14	0.41±0.16	−0.065	0.621	−0.065
	Total time	0.07±0.02	0.00±0.02	−0.01±0.02	0.01±0.02	−0.286	0.027*	−0.295
Steadiness	Number of errors	0.33±0.12	0.23±0.15	−0.01±0.09	−0.08±0.11	−0.317	0.014*	−0.328
Line tracing	Number of errors	0.18±0.05	0.11±0.05	0.09±0.04	−0.01±0.05	−0.313	0.015*	−0.324
	Total time	−0.02±0.02	−0.02±0.03	−0.09±0.03	−0.14±0.06	−0.324	0.012*	−0.336
Tapping	Number of hits	−0.07±0.01	−0.07±0.01	−0.07±0.01	−0.08±0.02	0.001	0.991	0.001

Average laterality indices (±SEM) for all parameters obtained for the aiming, steadiness, line tracing, and tapping tasks for the four age groups tested. Individual data (n = 60) were used to calculate the linear (Pearson) correlation coefficients between laterality indices and age for each parameter. Significance levels are given for p<0.05 (*). To obtain comparability of linear (Pearson) correlation coefficients, Fisher-Z values are given after Fisher-transformation.

#### Time course of hand dominance changes

While there was a general loss of right-hand dominance with increasing age, the average age at which changes became evident was dependent on the individual task and parameter. For example, the time course for change of the laterality indices for the total time in the aiming task confirmed that subjects in group 25 performed better with their right hand than with their left hand. The right-hand advantage was completely absent in the group 50 subjects. For this group, and for the subjects in groups 70 and 80, we observed a balanced performance between the left and right hands, implying that a shift of laterality is already present in middle age. In contrast, the laterality indices for the number of errors in the steadiness task showed a more gradual change with small shifts in laterality for the subjects in group 50. Only subjects in groups 70 and 80 showed a loss of right hand dominance.

The line-tracing task requires the subject to perform both accurately and as quickly as possible. Since there is a trade-off between speed and error rate, subjects were instructed to make as few errors as possible. The laterality indices for the number of errors and total time both showed a continuous decline with age beginning with group 50, indicating that right hand dominance progressively decreases. However, both parameters differed in that for the total time, a balanced performance with both hands was already evident in group 25 subjects. It is possible that subjects change their strategy with age, shifting from speed-oriented to error-minimizing behavior. However, we found that the reduction in errors with the left hand compared to the right was not achieved by older subjects taking more time to perform the task. On the contrary, the average time needed to complete the task was also shorter for the left hand than for the right hand. This behavior began to emerge in group 70 and continued in group 80. As a result, in the line tracing task, the progressively increasing dominance of the left hand with increasing age was reflected in both the error rates and the time needed for completion.

#### Less drastic impairment of non-dominant hand performance causes dexterity equalization

To identify possible contributing factors to shifts in hand dominance, we analyzed the age-related decline of the right and left hand performances separately for each task and parameter by normalizing the correlation coefficients with a Fisher-transformation and subtracted the Z-values (right-left). For all parameters that showed a strong equalization in performance with increasing age, the result of the subtraction was calculated by positive amounts (total time for the aiming task = 0.07, number of errors in the steadiness task = 0.025, number of errors and total time for the line tracing task = 0.013 and 0.024). This indicates that right-hand performance declines more with age than left-hand performance. Accordingly, the loss of right hand dominance appears to be largely due to the right hand being more sensitive to age-related alterations than the left hand. For the two factors which showed no equalization of hand performance, the subtraction of Z-values was also carried out (number of errors in the aiming task = −0.03, number of hits in the tapping task = 0.06).

### Experiment 2

#### Frequency of hand use in everyday tasks

**Figure 4:**
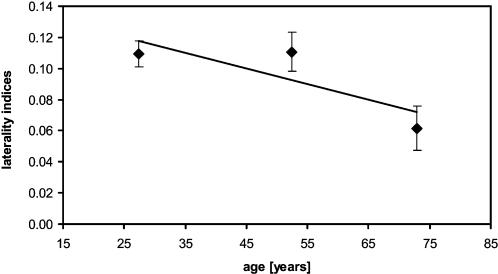
Changing hand use over the lifespan Illustration of age-dependent changes in the laterality indices (±SEM) obtained from acceleration measurements used to objectively assess the frequency of hand use (+1 for right hand superiority to –1 for left hand superiority) for all subjects tested (n = 36). There is a significant linear correlation of individual laterality indices with age (Pearson, r = −0.447, p = 0.007) indicative of a loss of right (dominant) hand advantage with age.

To address possible age related changes in hand use using objective measurements, 36 subjects agreed to wear acceleration sensors for several hours (average duration 3.12±0.41 h), at approximately the same time of day, during normal everyday indoor activities. Use of a computer was not allowed during this time (because more right hand use would be forced by the one-sidedness of the computer-mouse). The sensors were fixed on the wrists to detect acceleration of the hands during movements. As we were interested in left-right differences, we calculated the laterality index as described above for all subjects (**Tab. 2**). There was a significant correlation between the laterality indices of hand use and age (Pearson correlation, r = 0.447, p = 0.007). In the younger, group 25 subjects we found superiority in the frequency of dominant right hand use (laterality index: 0.11±0.01). The same observation was made in group 50 subjects (laterality index: 0.11±0.01). However, the group 70 subjects showed a more balanced frequency of dominant and non-dominant hand use (laterality index: 0.06±0.01) confirming a tendency towards equalization of hand use in older age during everyday activities (**Fig. 4**). There were no gender-specific differences in the laterality indices found for group 25 (F_(1,12)_ = 0.482, p = 0.502), group 50 (F_(1,8)_ = 0.293, p = 0.605), and group 70 (F_(1,13)_ = 0.703, p = 0.418).

**Table 2:**
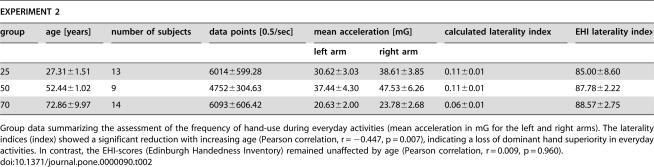
Frequency of hand use during everyday activities

EXPERIMENT 2							
group	age [years]	number of subjects	data points [0.5/sec]	mean acceleration [mG]		calculated laterality index	EHI laterality index
				left arm	right arm		
25	27.31±1.51	13	6014±599.28	30.62±3.03	38.61±3.85	0.11±0.01	85.00±8.60
50	52.44±1.02	9	4752±304.63	37.44±4.30	47.53±6.26	0.11±0.01	87.78±2.22
70	72.86±9.97	14	6093±606.42	20.63±2.00	23.78±2.68	0.06±0.01	88.57±2.75

Group data summarizing the assessment of the frequency of hand-use during everyday activities (mean acceleration in mG for the left and right arms). The laterality indices (index) showed a significant reduction with increasing age (Pearson correlation, r = −0.447, p = 0.007), indicating a loss of dominant hand superiority in everyday activities. In contrast, the EHI-scores (Edinburgh Handedness Inventory) remained unaffected by age (Pearson correlation, r = 0.009, p = 0.960).

## Discussion

### Age-related discrepancy between self-rated hand dominance and active performance

We obtained the EHI handedness-scores for the 96 subjects participating in experiments 1 and 2. There was clearly no correlation between the score and the age of the subjects, pointing towards an unchanged dominance of the right hand with increasing age (Linear Pearson-correlation, r = −0.041, p = 0.692). This outcome was not surprising, as strong right-hand dominance was part of the selection criteria.

In experiment 1, we compared self-rated hand dominance with the results of a fine motor test-series (MLS). The scores on the EHI and the actual hand performance collided, as 4 parameters indicated an equalization of hand dominance. In most of the remaining parameters, at least a trend towards hand equalization could be demonstrated. A Pearson correlation of the EHI scores and MLS laterality indices confirmed that both measures were unrelated (r = 0.130, p = 0.322).

In experiment 2, self-rated hand dominance was compared with the sensor-based assessment of hand-use in everyday living. Similar to the results of experiment 1, the calculated laterality indices for hand-use indicate a loss of right hand dominance with increasing age, but also showed no significant correlation with the obtained EHI-scores (r = 0.050, p = 0.771).

**Figure 5:**
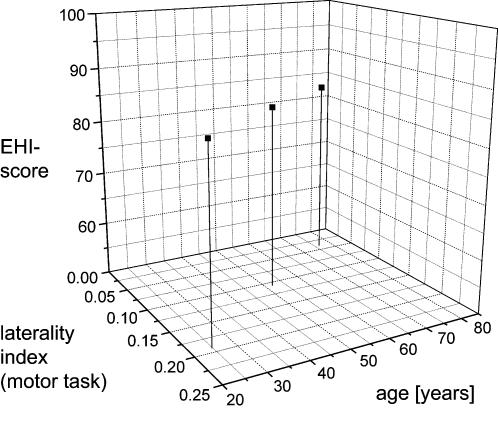
Contradictory results of questionnaires and practical tasks Three dimensional plot illustrating the age-dependence of the sensitivity of the EHI-scores obtained from the Edinburgh Handedness Inventory, and the laterality indices averaged over all motor tasks and parameters (laterality of motor performance) for the 4 age groups investigated. While subjects of all age groups are characterized by approximately the same handedness score (EHI ≥ 70), there is a distinct reduction in laterality, indicating that the age-related loss of dominant hand advantage remains largely unrealized by the subjects.

We conclude that although the older subjects were unaware of changes in their handedness, a clear trend towards an equalization of hand performance under controlled (Experiment 1, **Fig. 5**) and everyday conditions (Experiment 2, **Fig. 4**) could be measured. Our results indicate that hand dominance is affected during the normal aging process. The significant correlation between subjective hand preference and task performance present in young subjects [23], [24], [25] disappears with aging. In contrast, while older subjects subjectively reported a strong right-hand preference, a variety of motor tasks and measurements of the frequency of hand use during everyday activities indicate a trend towards equalization of left and right hand use, and of the quality of hand performance with increasing age.

### General influence of aging

As expected, we found a decline of the level of fine motor performance with age, regardless of whether the tests were performed with the right or the left hand. The effect of aging on the capacity to perform fine motor movements is well-established in the experimental literature [29], and the extent of age-related slowing of hand-movements is positively correlated with task-difficulty (coarse versus fine motor performance) [30], [31].

The age related-decline may be the result of a general slowing down of central cognitive processes [29], [32], [33] which is assumed to affect the motor performance of less coordinated repetitive movements, like those in a tapping task [34], [35]. Whether cognitive processing is a leading global factor of the nonspecific slowing down, or if it acts selectively on different aspects of the movement process, is still under debate. Experimental evidence points towards the necessity of separating different movement stages, such as planning and execution, and provide evidence that these are differentially sensitive to age. Specifically, the movement-planning stage is prolonged, and the start of movements is delayed relative to the go signal [36]. But the actual movement seems affected by age as well, as motion trajectories by elderly people in aiming tasks seem to be less linear and more irregular than in younger subjects [36], and compensatory movements in pursuit-tracking experiments, at least at high speeds of the target, are slower and more variable [37].

### Changes in hand dominance with age

Apart from replicating the recognized age-related decline, we provide evidence that the decline is greater for the right hand, which shifts the manual dexterity towards a more balanced performance with both hands. These results were obtained under highly controlled experimental conditions, and are supported by recording hand use during everyday activities. This demonstrates that, in contrast to younger subjects, the elderly employ both hands with equal frequency. Interestingly, this completely contradicted the results of the common EHI-questionnaire, which showed no change in self-rated handedness over the lifespan. This was a somewhat surprising result, because previous studies in younger subjects revealed that self-rated handedness and the level of motor performance show significant coherence [23], [24], [25].

### Cortical correlates of hand dominance

Handedness is known to be correlated with specific lateral asymmetries; for example, higher cortical excitability in the dominant hemisphere for right-handers that are absent in left-handed-subjects [38], and anatomic asymmetries such as deeper left central sulci that are highly correlated with the degree of handedness in male subjects [39]. If handedness changes with age, as our results indicate, this may be due to asymmetric changes in the hemispheres rather than reflecting long-time plastic changes.

The number of brain areas and the intensity of their activation in motor tasks of the upper extremities changes during the human aging process; for example, greater cortical and more pronounced bilateral activation of sensorimotor regions in EEG recordings in elderly subjects [40], or a greater activation in the contralateral sensorimotor cortex, lateral premotor area, and ipsilateral cerebellum in a fMRI study [41], [42]. The increase in cortical activation with aging is not specific for movement tasks, but is also commonly found in studies addressing visual and mnemonic abilities [43], [44], [45]. These are usually interpreted in terms of recruitment of additional brain areas to provide compensatory processes, or regarded as evidence for reorganization and redistribution of functional networks thereby compensating for age-related structural and neurochemical changes [41]. The common finding of increased bilateral activation and additional activation in the frontocentral cortex (supplementary motor area region) lead to the conclusion that there is an increased need for cognitive control of simple motor tasks in old age [40].

Frontal activity during cognitive performance seems to be less lateralized in the elderly (hemispheric asymmetry reduction in older adults, HAROLD; for a detailed review, see [46], [47] compared to young adults which is supported by functional neuroimaging studies in the domains of working memory [48], perception [49], and inhibitory control [50] and may reflect functional dedifferentiation or more likely, a form of compensation [45]. With the exception of frontal activity, these common findings of age-related changes may provide an explanation for changes in the level of performance with age – like the decrease in movement performance replicated in our data – but do not help to resolve the puzzling tendency toward ambidexterity. Is there any evidence for asymmetric aging of the different hemispheres?

Historically, changes in lateral asymmetry with aging were often reported, and proposed to constitute a basic phenomenon reflecting compensational adaptations in response to age-related changes in neural processing. Some authors assume that the right hemisphere shows a larger age-related decline (called the right hemi-aging model) than the left hemisphere [51], [52]. This assumption was promoted by behavioral studies in the domains of cognitive and sensorimotor processing [53], [54] and interpreted assuming a faster age-related decline in the right hemisphere in terms of blood flow, neuron-death, and other physiological changes [55]. In contrast, other authors have argued the opposite [56], as left-hemispheric functions were believed to be better preserved because they are practiced more intensively than right-hemispheric functions [57]. Recent anatomical studies investigating structural changes that occur during the normal aging of primate cerebral hemispheres (for a review see [58]) disproved assumptions such as a major neuron loss with aging, and showed no asymmetrical developments at all [59].

If neurophysiological evidence does not support asymmetric aging of the hemispheres, a possible explanation for the move towards ambidexterity in upper limb movements with age comes from the concept of use-dependent plasticity. The advantage of the dominant hand is determined early in life, and is intensified by practice through everyday activities. When these activities decrease after retirement, or by the limitations in older age and sedentary lifestyles [16], [60], [61], it is conceivable that the practice-based superior performance of the right hand is no longer maintained, thus approaching the performance level of the left hand. This is in line with the more balanced use of both hands in everyday-life of aged subjects we found. In particular, fine motor abilities, which undergo extreme changes during the aging process, seem to depend on intact physical fitness [1]. That the age-related lateral equalization in motor performance depends on physical abilities is also in accordance with the comparable equalization in cognitive performance, as many studies indicated a correlation between age-related changes in physical fitness and cognitive performance [13], [14], [62].

### Questionnaires versus practical performance

In contrast to the findings presented here, several older studies have suggested that right-handedness might increase with age [63], [64], [65]. This view was largely based on the theory of asymmetrical aging of the cortical hemispheres ([51], [53]; for a sex-specific account of age-related changes of visual field asymmetries, see [66]), especially a more rapid aging of the right hemisphere [67], which is not supported by more recent neurophysiological evidence [58]. It should be emphasized that the empirical evidence for an increase in handedness stems, at least partly, from the use of questionnaires [64]. Our data clearly show that questionnaires and self-rating do not estimate actual performance in elderly subjects. As self-ratings provide a reliable tool for the assessment of handedness in young subjects [68], the discrepancy between self-rated handedness and movement data for older subjects may seem puzzling. On the other hand, it has been hypothesized that the elderly are prone to extreme decisions [69], as they show a response bias toward choosing the extreme end of a scale continuum. This bias could provide an explanation for why self-rated handedness increases with age, both in the literature and in our data. However, data from actual movement tasks in our study do not support a shift towards increasing right-handedness with age, as we found severe discrepancies between the results from the Edinburgh Handedness Inventory and practical tasks assessing motor performance with increasing age.

### Summary

The use of practical tasks and the recording of hand use during everyday activities shows that the differences in the performance of the dominant and non-dominant hands which are clearly present at young age, diminish with increasing age. Apart from replicating the common finding of a decline in fine motor performance, our results provide evidence that handedness may become more balanced between early and late adulthood.

Self-ratings (EHI) indicate that the elderly subjects were unaware of these changes, i.e. the fact that hand performance had become more balanced. While at younger ages the outcomes of the self-rating and performance measures yielded a high coherence, the lack of awareness of their own performance level imposes severe restrictions on the use of questionnaires for handedness assessments in advanced age groups. As neurophysiological studies do not support a clear neural basis for this finding of changes in laterality, our results provide evidence for the importance of the concept of use-dependent plasticity.
